# Induction of Female-to-Male Sex Change in Adult Zebrafish by Aromatase Inhibitor Treatment

**DOI:** 10.1038/srep03400

**Published:** 2013-12-02

**Authors:** Kanae Takatsu, Kaori Miyaoku, Shimi Rani Roy, Yuki Murono, Tomohiro Sago, Hideyuki Itagaki, Masaru Nakamura, Toshinobu Tokumoto

**Affiliations:** 1 Department of Biology, Faculty of Science, National University Corporation Shizuoka University, Oya 836, Suruga-ku, Shizuoka 422-8529, Japan; 2 Integrated Bioscience Section, Graduate School of Science and Technology, National University Corporation Shizuoka University, Ohya 836, Suruga-ku, Shizuoka 422–8529, Japan; 3 Department of, Faculty of Education, National University Corporation Shizuoka University, Oya 836, Suruga-ku, Shizuoka 422-8529, Japan; 4 Okinawa Churashima Foundation, 888 Ishikawa Motobu-cho, Okinawa 905-0206, Japan

## Abstract

This study investigated whether undifferentiated germ and/or somatic stem cells remain in the differentiated ovary of a species that does not undergo sex changes under natural conditions and retain their sexual plasticity. The effect of aromatase inhibitor (AI)-treatment on sexually mature female zebrafish was examined. A 5-month AI treatment caused retraction of the ovaries after which testes-like organs appeared, and cyst structures filled with spermatozoa-like cells were observed in sections of these tissues. Electron microscopic observations revealed that these cells appeared as large sperm heads without tails. Sperm formation was re-examined after changing the diet to an AI-free food. A large number of normal sperm were obtained after eight weeks, and no formation of ovarian tissue was observed. Artificial fertilization using sperm from the sex-changed females was successful. These results demonstrated that sex plasticity remains in the mature ovaries of this species.

In fish, the sex reversal of reproductive organs can be induced by treatment with sex steroids during sexual differentiation in juveniles. This experimentally induced sex change was first described in medaka^1^, and artificial sex change has been intensively studied since^2^. Although these experiments demonstrated that gonadal cells and somatic cells in the gonads possess cell type plasticity, it was not possible to induce sex change using sex steroids following sex differentiation. Thus, it was thought that sexual plasticity is lost after sex differentiation.

Female-to-male sex change is associated with a decrease in estrogen levels, followed by an increase in androgen levels^3^. Estrogens are produced by the conversion of aromatizable androgens by cytochrome P450 aromatase (P450arom), and the actions of P450arom are essential for sex differentiation and ovarian development in fish and other vertebrates^4^,^5^. Recently, it has become possible to reduce estrogen synthesis by inhibiting this aromatase activity using non-steroidal aromatase inhibitors (AIs), such as fadrozole hydrochloride, which is a reversible competitive inhibitor^6^. Further studies showed that sex change could be induced in many types of fish by aromatase inhibitor (AI) treatment during sex differentiation. In Japanese flounder (*Paralichthys olivaceus*) and tilapia (*Oreochromis niloticus*), brief treatment with AI during sex differentiation causes a sex reversal in which genetic females develop into phenotypically normal males^7^,^8^.

Undifferentiated ovary-like gonads are initially developed during gonadal development in juvenile zebrafish, regardless of genotypic sex^9^. In genotypic male zebrafish, all oocytes disappear from the gonad by 30 days post-hatching, and spermatocytes develop concomitant with testicular differentiation^10^. In contrast, oocytes in the female ovaries continue to grow to maturation. The phenomenon of the presence of undifferentiated ovary-like gonads during the juvenile period is known as juvenile hermaphroditism. Recently, it has been reported that the massive, male-specific disappearance of oocytes from the gonad during the transition from ovary-like tissue to testis tissue is caused by apoptosis^11^. Therefore, it was suggested that oocyte apoptosis plays a significant role in the testicular differentiation of juvenile zebrafish. In zebrafish, the gonadal masculinization of genetic juvenile females can be induced by the dietary administration of an AI (fadrozole)^12^. Recently, two independent studies provided evidence for a population of undifferentiated stem cells in the ovary of adult zebrafish that can produce both types of gametes^13^,^14^.

In this study, we examined whether AI (fadrozole) treatment induces a sex change in adult zebrafish. Our results support the hypothesis that sexual plasticity persists in adult zebrafish following sex differentiation, indicating that undifferentiated stem cells are maintained in adult fish that do not undergo sex change under natural conditions.

## Results

### Ovarian fluorescent and transparent transgenic zebrafish lines

**Figure 1:**
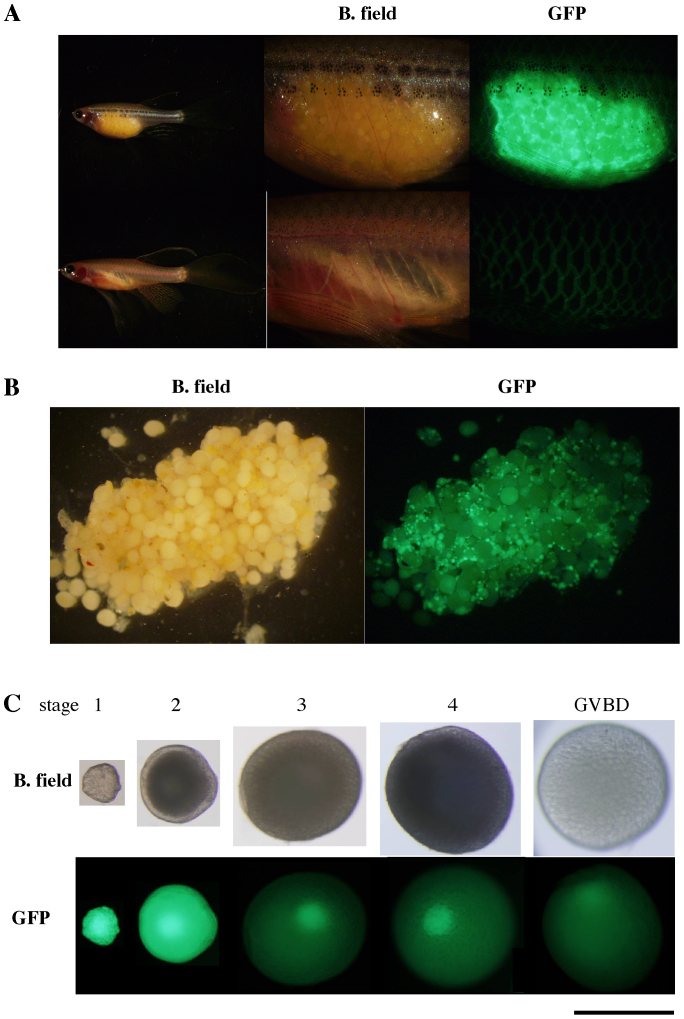
Ovarian fluorescent and transparent transgenic zebrafish. (A) The left panels show the full view of adult female and male *TG(β-actin:EGFP);roy*, respectively. Side views taken under brightfield and GFP filter views of the trunk are shown. (B) An ovary dissected from transgenic zebrafish, in brightfield and GFP filter views. (C) Follicle-enclosed oocytes in various stages (1 to 4) and after germinal vesicle breakdown (GVBD) were separated from the ovary. The scale bar indicates 500 μm.

To allow us to monitor the changes in the ovaries of living fish during fadrozole administration, we used ovarian fluorescent and transparent transgenic zebrafish lines. The transparent strain of zebrafish, *roy*, is deficient in the production of iridescent color by iridophores, and the viscera and reproductive organs are thus visible from the outside of the fish body (Figure 1A). In the transgenic (*TG*) line with ovarian fluorescence, GFP is loosely bound to the nucleus (germinal vesicles); thus, the germinal vesicles fluoresce bright green. As this fluorescence is relatively strong in small oocytes at stages 1 and 2 (Figure 1B and C), this property was well suited for monitoring ovarian retraction.

### Morphological changes in ovaries

**Figure 2:**
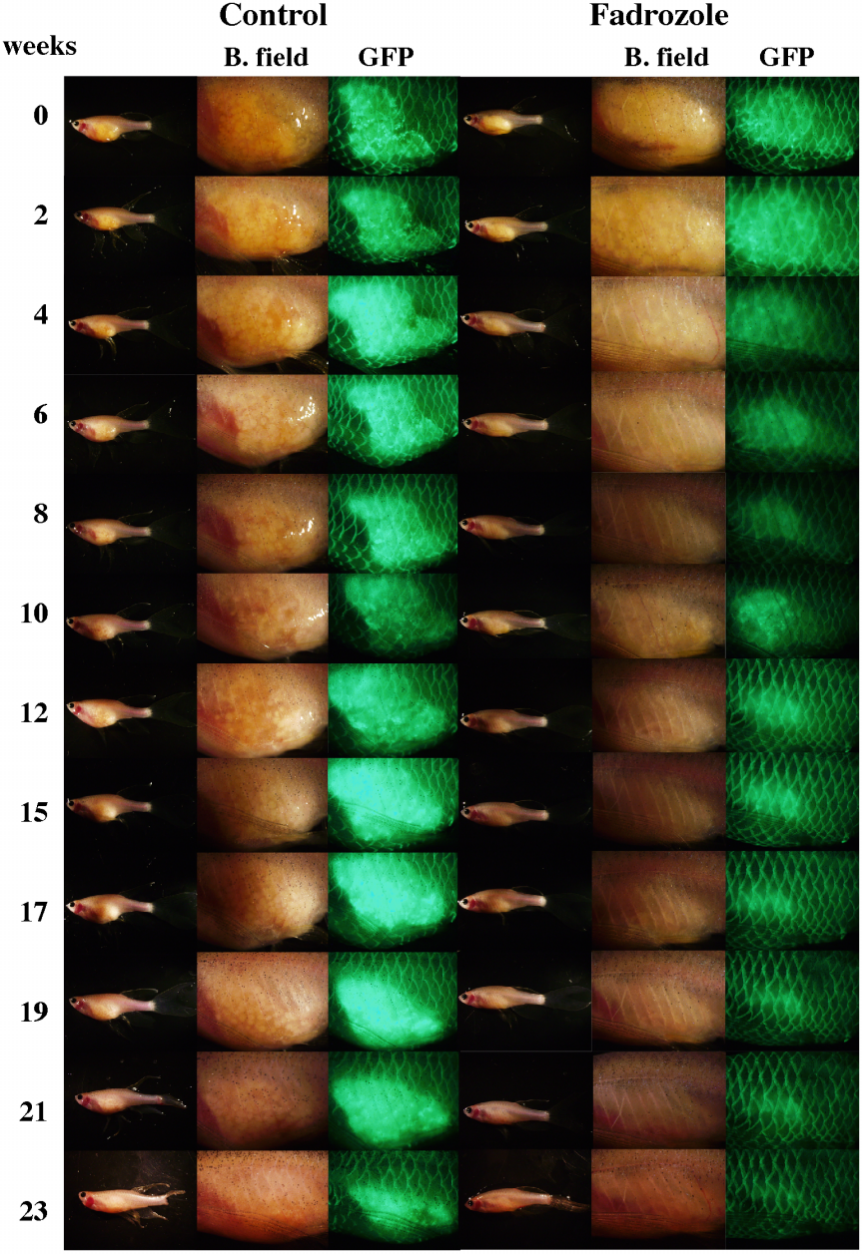
The effect of fadrozole treatment on sexually mature female zebrafish. The morphology of ovaries during the treatment was monitored by fluorescent observations at 2-week intervals. Photographs of whole fish and ovarian tissues under brightfield (B. field) and GFP filter views (GFP) are shown.

**Figure 3:**
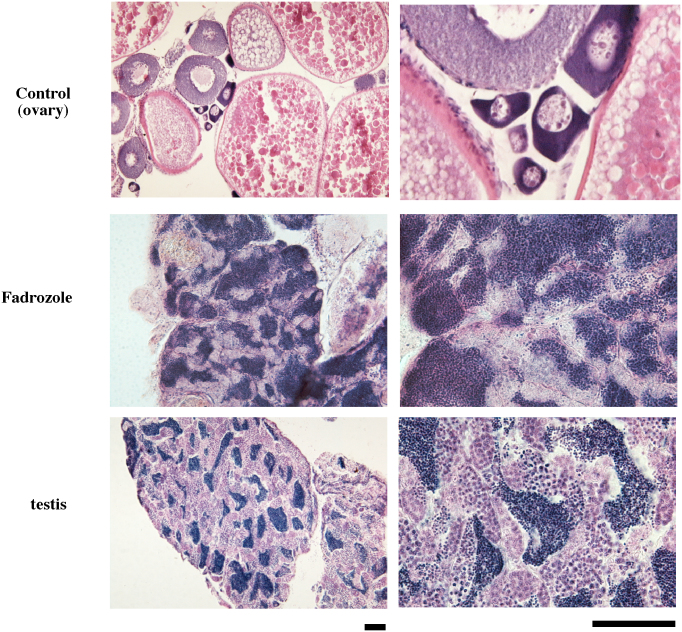
Testis-like tissues formed in fadrozole-treated females. Sections of ovarian tissue from control-treated female (Control), testis-like tissues from fadrozole-treated female (Fadrozole), and testis from a normal male (testis) are shown. The scale bars indicate 100 μm.

**Figure 4:**
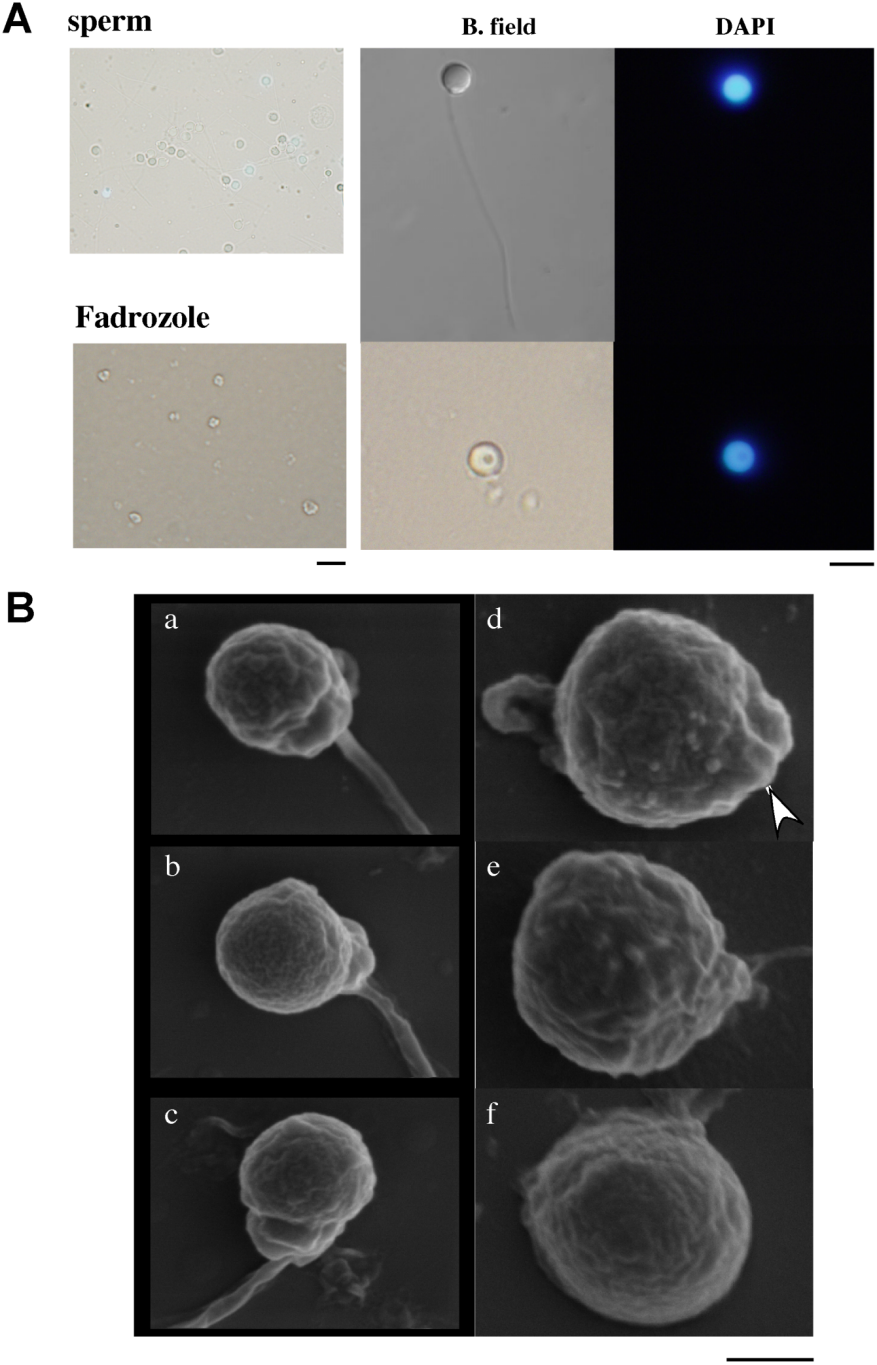
Spermatocyte-like cells found in the testis-like tissues formed in a fadrozole-treated female. (A) Cells were collected from minced tissues and observed after staining with DAPI. Sperm from normal testis (sperm) and spermatocyte-like cells from a fadrozole-treated female (Fadrozole) in brightfield (B. field) and DAPI filter views. The scale bar indicates 10 μm. (B) Electron microscopic observation of spermatocyte-like cells. The cells were fixed in 4% paraformaldehyde in phosphate-buffered saline (pH 7.6) and washed three times with de-ionized water. The cells were air-dried and observed under a scanning electron microscope (JSM6300, JEOL Ltd., Tokyo, Japan). The sperm from normal testis (a–c) and spermatocyte-like cells from a fadrozole-treated female (d–f) are shown. The arrowhead in panel d indicates a mid piece-like structure. The scale bar indicates 1 μm.

**Figure 5:**
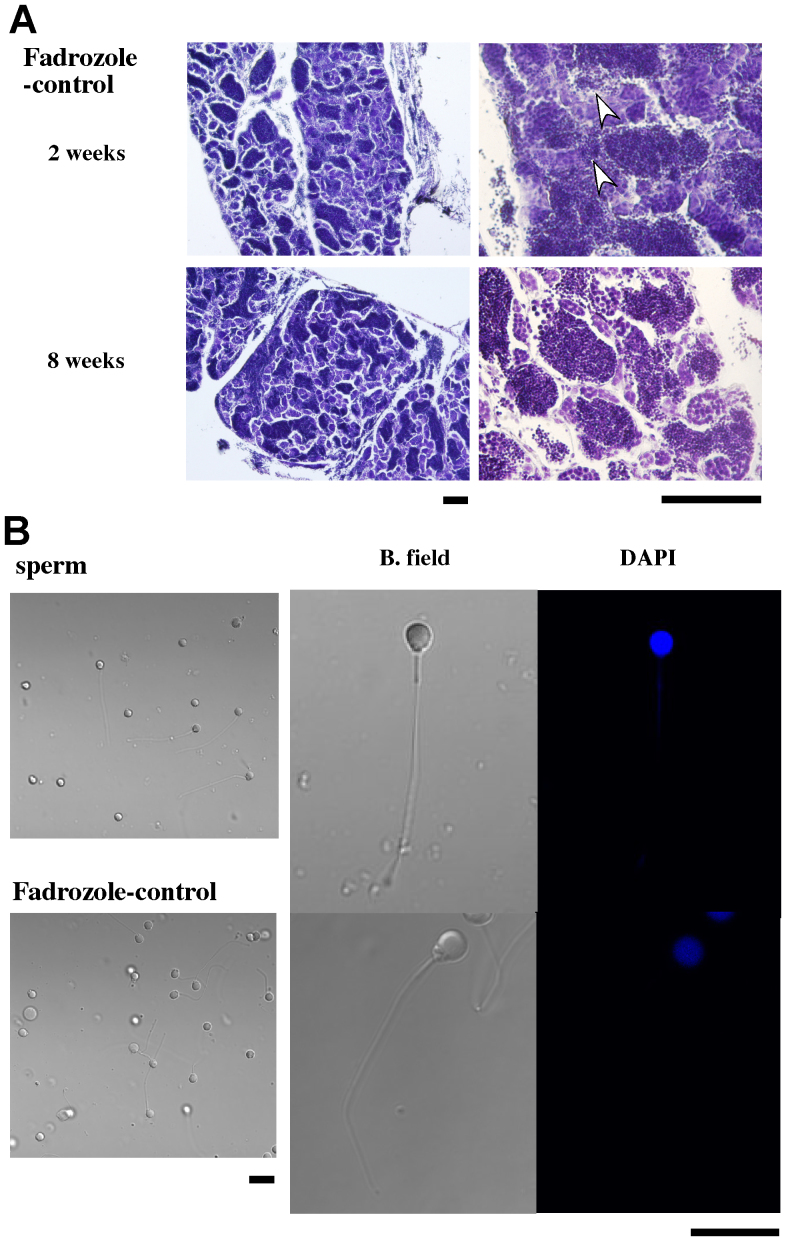
Normal sperm formation following prolonged fadrozole-free treatment after testis-like tissue formation. (A) Sections of testis-like tissue from females at 2 weeks or 8 weeks of control treatment after fadrozole treatment (Fadrozole-control) are shown. Dispersed spermatozoa-like cells are indicated by white arrowheads. The scale bars indicate 100 μm. (B) Cells were collected from minced tissues and observed after staining with DAPI. Sperm from normal testis (sperm) and a fadrozole-control-treated female (Fadrozole-control) in brightfield (B. field) and DAPI filter views. The scale bar indicates 10 μm.

**Figure 6:**
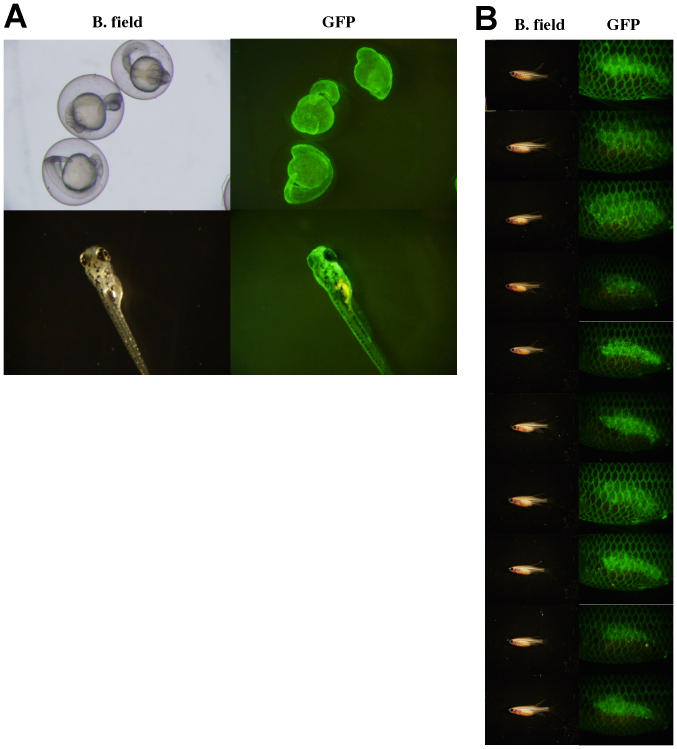
Juveniles developed by the artificial fertilization of eggs from a normal female with sperm from a sex-changed female. Embryos at 24 (upper) and 48 hours (lower) (A) and ten embryos at approximately one month after fertilization (B) in brightfield (B. field) and DAPI filter views.

Although ovary size was reduced within several months, even in fish under the control conditions in a group of females alone (27%, n = 11), the ovaries underwent oscillatory changes in size during the treatment. The oscillatory intervals were approximately two weeks but were not constant among individuals. The ovaries of the fadrozole-treated fish gradually reduced in size. After five months, the ovaries retracted completely, and the fluorescence-expressing oocytes had disappeared in all fish (100%, n = 9) (Figure 2). Testis-like white tissues were observed in several fish (Supplementary Fig. S1), and mating and/or *in vitro* fertilization experiments were conducted using these fish to examine whether normal testis had developed in these fish. Although this trial was conducted more than three times for each fish, no fertilized egg was obtained. The tissues in the control and experimental groups were then subjected to histochemical examination (Supplementary Fig. S2 and Figure 3). The morphology of ovarian tissues in the control fish showed no significant change compared to the ovaries from untreated females (Figure 3). However, cysts filled with spermatozoa-like cells were observed in the fadrozole-treated fish; the cells were a consistent size but larger than the sperm cells observed in normal males. The cells were extracted from the testis-like tissues, and sperm formation was examined by fluorescence microscopy or electron microscopy. A morphology of spermatozoa-like cells without tails was observed under fluorescence microscopy (Figure 4A), and electron microscopic observations revealed large sperm heads without tails. A midpiece-like structure was observed in some of the spermatozoa-like cells (Figure 4B). We then evaluated the effect of discontinuing the AI treatment after the formation of the testis-like tissues and re-examined sperm formation. At two weeks after treatment discontinuation, broken cyst structures and dispersed spermatozoa-like cells were observed (Figure 5A). Then, cyst structures filled with different stages of spermatozoa-like cells, similar to those observed in the normal testis, were observed in sections from fadrozole-treated fish at 8 weeks after replacing the AI food with the control treatment. As shown in Figure 5B, a large number of normal sperm was extracted from the AI-treated testis-like tissues. Artificial fertilization using these sperm and eggs from a non-transgenic female was successful, and juveniles developed normally (Figure 6). Fluorescence microscopy analysis confirmed that the juveniles had developed from the sperm formed in the sex-changed female. As expected, the juveniles were all females (Figure 6B), and ovaries with green fluorescence were observed, except one in which the reproductive tissue had not yet developed at one month (one of 18). Again, this result indicates that the sperm from the sex-changed female were capable of fertilization.

## Discussion

One of the amazing reproduction system phenomena, sex change, has been reported in such teleost fish as cichlids and tropical fish. Sex change is a purely ontogenetic event in some species but can be triggered in others species by environmental stimuli, such as interactions with conspecifics. The size advantage model for sequential hermaphroditism remains the most widely accepted evolutionary explanation of the adaptive significance of sex change^15^,^16^,^17^. This model posits that, if an individual can reproduce more effectively as one sex when small or young and as the other sex when larger or older, it should change sex at some point in its life history. For example, sex change follows the size-advantage model in Nile tilapia: these fish can change sex from female to male before forming the nest.

Additionally, sex reversal, i.e., the development of an ovary or testis independent of the genetic sex, can be induced in juvenile fish by treatment with sex steroids. The experimental induction of sex reversal in species that do not undergo sex change in nature was first reported in medaka^1^. Indeed, the administration of sex steroids prior to gonadal formation induced all-female or all-male gonads according to the type of steroids administered^2^. The same results have been reported in other species, including zebrafish^12^.

In this study, we tested the possibility that the capacity for sex change remained in the adult fish of species that do not undergo sex change in nature. Specifically, we examined whether undifferentiated germ and/or somatic stem cells are present in the differentiated ovary in such species and examined the effect of AI treatment in sexually mature female zebrafish. In all the females tested, AI treatment caused ovarian retraction, followed by the development of testes-like organs. Cyst structures filled with spermatocyte-like cells were observed in the sections of these organs, and electron microscopic observation revealed that the cysts contained large sperm heads without tails.

It has been reported that estradiol-17β (E2) plays an important role in the final differentiation of sperm^18^. Furthermore, zebrafish treated with low concentrations of tributyltin developed abnormal sperm without tails^19^, an effect was thought to be caused by the disruption of E2 activity. The same effect has been reported for aromatase inhibitor treatment^20^. Based on these findings, the results in the present study can be interpreted as follows: a significant decrease in endogenous E2 levels triggered the retraction of the ovary and induced testis formation, though the final differentiation of the sperm was inhibited due to the extremely low concentration of E2. The plasma E2 levels were 1.35 ng/ml (±0.6, n = 3) in the control group but under the limit of detection in the AI-treated fish. Thus, functional sperm were not produced by fish that were exposed to continuous AI treatment. However, normal and fertilization-competent sperm were formed after replacing the AI-containing food with an AI-free food. Our results demonstrated that undifferentiated germ stem cells persist in adult fish, similar to the results obtained in tilapia and medaka^21^. The testis appeared to have developed from the area near the cloaca rather than the ovarian tissue (Supplementary Fig. S1). Indeed, it is thought that a small number of undifferentiated germ stem cells, which serve as the source of the spermatogenic cells in the sex-reversed fish (Supplementary Fig. S1), may remain in this area. Future detailed analyses of the retraction of the ovary and formation of the testis should lead to the identification of stem cells. Ample evidence for the presence of undifferentiated stem cells in the adult female zebrafish has been reported previously. Dranow *et al* showed that adult zebrafish could be sex reversed to fully functional males following the depletion of most of their germ cells^14^. Wong *et al* also reported sex reversal in the zebrafish by the transplantation of female germ cells into a male^13^. Fertile sperm were produced from the transplanted germ cells. These results suggest that there is a population of undifferentiated stem cells remaining in the adult female zebrafish.

We were also interested in investigating whether the sex-changed females would engage in reproductive behavior with normal females. Thus, we attempted to pair sex-changed and normal females. Although we used females with ovulated oocytes, the sex-changed female did not show any behavior toward the normal female. This result suggested that the sex change induced in this study was restricted to the gonadal tissues and did not cause changes in the brain. However, as normal males from the same batch as the females treated in this study also failed to show any mating behavior, this result may be attributed to the age of the fish (2 years old at the end of the AI treatment). To address the possibility of changes in the brain, these experiments should be repeated with younger fish.

## Methods

### Animals

The transgenic line *TG (β-actin:EGFP)* was established by Hsiao et al^22^,^23^. Although the cDNA integrated in this strain was constructed for the expression of *EGFP* driven by the medaka *β-actin* promoter, the expression of *EGFP* is restricted to the oocytes and gills in adult fish. A *roy* mutant zebrafish, which is deficient in the production of iridescent color exhibited by iridophores, resulting in a transparent body, was isolated. We crossed the *roy* and *TG* strains to establish a strain that enables the direct observation of oocytes in living fish. The resulting strain, *TG* (*β-actin:EGFP);roy*, is highly transparent, and its oocytes are easily observed by fluorescence in living fish. The *TG* zebrafish were bred and maintained at 28.5°C and a 14-h light/10-h dark cycle^24^. All experiments using zebrafish were carried out with the approval from the institutional ethics committee of Shizuoka University, Japan, strictly following the guidelines set for the usage of animals by this committee.

### Reagents

17,20β-DHP and DES were purchased from Sigma Chemical Co. (St. Louis, MO, USA). Fadrozole was obtained from Wako Pure Chemical Industries (Osaka, Japan).

### Hormone preparation and treatments

Fadrozole was mixed with powdered fish food grains (Chroma, Kobe, Japan) and kept at room temperature in a dark box. The sexually mature females used for treatments were selected after checking for maturity, as confirmed by the observation of normal mating behavior or by artificial fertilization using a recently developed technique for the induction of ovulation^25^. The fish were divided into control and fadrozole treatment groups of 7–10 fish each. Each group was housed in separate small aquarium with a filtration system placed in an incubator set at 28.5°C and a 14-h light/10-h dark cycle. The fish were fed food containing fadrozole or not containing fadrozole twice a day. The amount of fadrozole used (0.2 mg/g diet) was selected based on the results of pilot experiments.

Ovarian morphology was monitored by fluorescence microscopy observations at 2-week intervals during the treatment period. Following anaesthetization with 0.5% tricaine, the ovaries were photographed under a binocular microscope under both brightfield and fluorescent lighting conditions.

### Fertility check

The fadrozole-treated individuals were allowed to mate with normal females. Each mating pair was housed in a plastic case paved with glass beads from evening until the morning of the next day. If no egg was obtained, ovulation was induced by adding a maturation-inducing steroid into the water^25^. Artificial fertilization was then conducted using squeezed eggs according to standard methods^24^.

### Sample collection

When testes-like organs were observed after the retraction of the ovaries, fish from the fadrozole-treated and control groups were sacrificed, and blood and gonad samples were collected after dissection. Blood samples were obtained from the heart using a 10-μl heparinized glass needle (tapered using a needle puller; Terumo, Fujinomiya, Japan) kept on ice^26^. The blood samples were centrifuged at 5,000 rpm for 10 minutes, and the plasma was collected from each sample separately and stored at −20°C until further analysis. The gonads were then carefully removed, fixed in Bouin's solution for 18 hours and then preserved in 70% ethanol for further analysis. The fixed samples were dehydrated in a graded ethanol series and embedded in paraffin. Ten-micrometer sections were prepared, and standard histological techniques were used to stain the gonad sections with hematoxylin and eosin.

### ELISA

The E2 levels in the plasma were measured using an ELISA kit according to the manufacturer's instructions (Cayman Chemical Company, Ann Arbor, MI, USA).

## Author Contributions

T.T., K.T. and K.M. designed the biological research, and K.T., K.M., S.R. and Y.M. performed the biological research. T.S. and H.I. performed the electron microscopic observations. T.T. and M.N. wrote the paper.

## Supplementary Material

Supplementary Information Dataset 1
