# Supplementary material for: Induction of Female-to-Male Sex Change in Adult Zebrafish by Aromatase Inhibitor Treatment
Source: Sci Rep. 2013 Dec 2;3:3400. doi: 10.1038/srep03400 (PMC3844967; doi:10.1038/srep03400)

**Supplementary Information for**

**Induction of Female-to-Male Sex Change in Adult Zebrafish by Aromatase Inhibitor Treatment.**

**Kanae Takatsu^1^, Kaori Miyaoku^1^, Shimi Rani Roy^2^, Yuki Murono^1^, Tomohiro Sago^3^, Hideyuki Itagaki^3^, Masaru Nakamura^4^, Toshinobu Tokumoto^1, 2^**

^1^Department of Biology, Faculty of Science, National University Corporation Shizuoka University, Oya 836, Suruga-ku, Shizuoka 422-8529, Japan

^2^Integrated Bioscience Section, Graduate School of Science and Technology, National University Corporation Shizuoka University, Ohya 836, Suruga-ku, Shizuoka 422–8529, Japan

**^3^**Department of, Faculty of Education, National University Corporation Shizuoka University, Oya 836, Suruga-ku, Shizuoka 422-8529, Japan

**^4^**Okinawa Churashima Foundation, 888 Ishikawa Motobu-cho, Okinawa 905-0206, Japan

Correspondence and requests for materials should be addressed to T.T. ([**sbttoku@ipc.shizuoka.ac.jp**](mailto:charles@darwin.co.uk))

**Figure caption of Supplementary Figures**

**Supplementary Figure. S1 Testis-like tissues formed from the area near cloaca.** Serial transverse sections around cloaca of fadrozole-treated female (about at four months treatment) were prepared. Photographs of sections are indicated in the order from posterior to anterior. Photographs of just before and after the cloaca are shown. Representative testis-like tissues are indicated by white arrowhead. The scale bar indicates 100 μm.


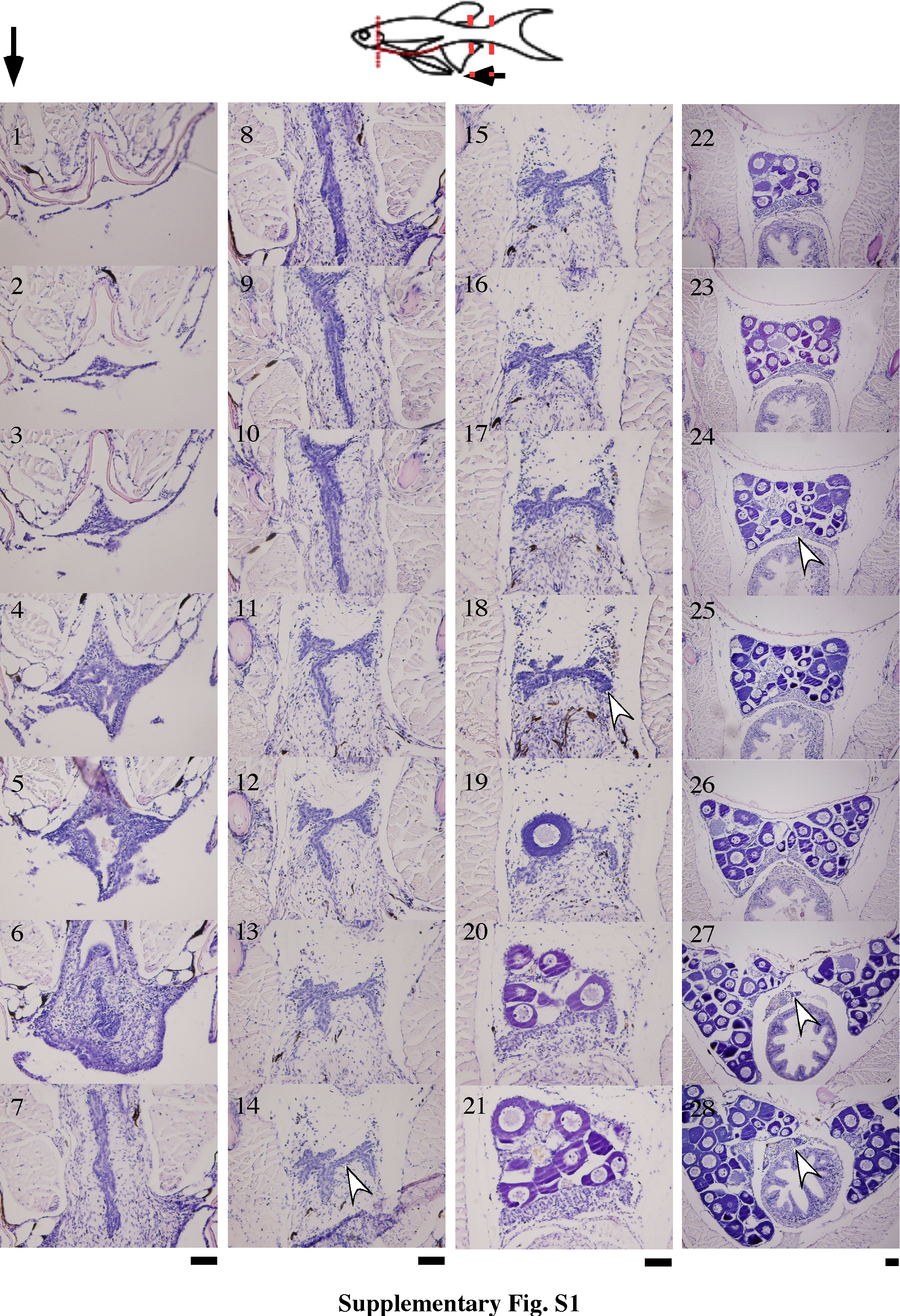


**Supplementary Figure. S2** **Testis-like tissues formed in a fadrozole-treated female.**

Testis or testis-like tissues were collected after dissection of normal male (testis), fadrozole-treated (Fadrozole) and fadrozole-control-treated female (Fadrozole-control).


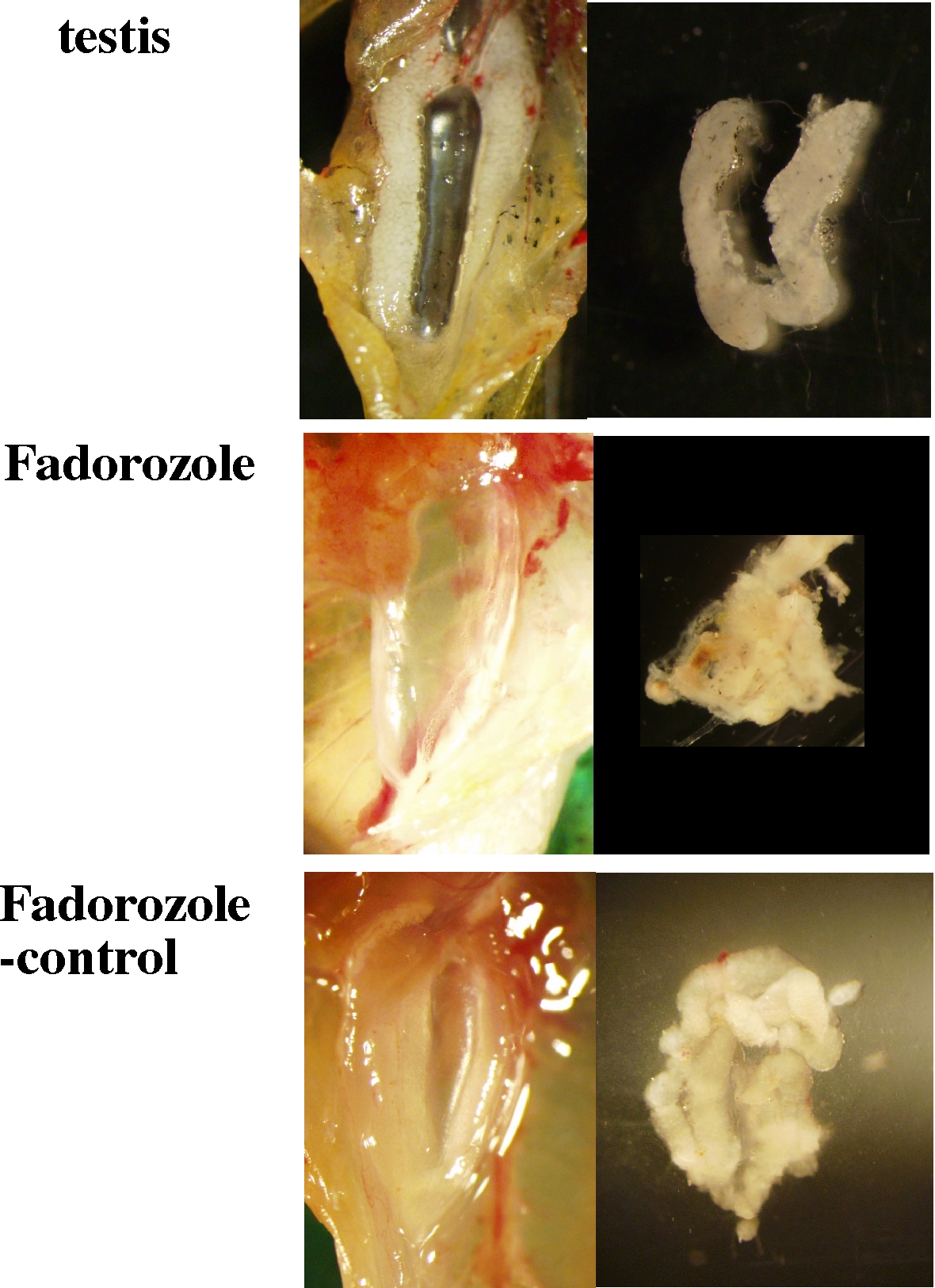

Supplement: Supplementary Information — Dataset 1 [file srep03400-s1.doc]
